# Harmonizing national abortion and pregnancy prevention laws and policies for sexual violence survivors with the Maputo Protocol: proceedings of a 2016 regional technical meeting in sub-Saharan Africa

**DOI:** 10.1186/s12919-018-0101-5

**Published:** 2018-04-24

**Authors:** Jill Thompson, Chi-Chi Undie, Avni Amin, Brooke Ronald Johnson, Rajat Khosla, Leopold Ouedraogo, Triphonie Nkurunziza, Sara Rich, Elizabeth Westley, Melissa Garcia, Harriet Birungi, Ian Askew

**Affiliations:** 1 Public Counsel, 610 South Ardmore Avenue, Los Angeles, CA 90005 USA; 2 Population Council, P.O. Box 17643-00500, Nairobi, Kenya; 3 Department of Reproductive Health and Research, World Health Organization, Avenue Appia 20, 1211, 27 Geneva, Switzerland; 4 Regional Office for Africa, World Health Organization, BP 06 Brazzaville, Republic of Congo; 5 Women’s Refugee Commission, 15 West 37th Street, 9th Floor, New York, NY 10018 USA; 6 International Consortium for Emergency Contraception (hosted by Management Sciences for Health), 45 Broadway, Suite 320, New York, NY 10006 USA

## Abstract

In April 2016, the Population Council, in partnership with the World Health Organization (WHO) and the International Consortium for Emergency Contraception, convened a regional meeting in Lusaka, Zambia, geared toward supporting countries in East and Southern Africa in meeting their obligations under the Maputo Protocol. These obligations include expanding access to women’s reproductive health services – especially women survivors of sexual violence. Government and civil society representatives from six countries participated: Botswana, Ethiopia, Kenya, Malawi, Rwanda, and Zambia. Countries were selected based on to their being priority settings for the projects that sponsored the meeting, coupled with the fact that they were each far enough along in addressing post-rape care to be able to develop concrete policy, programming, and/or legal action plans by the end of the meeting.

The meeting was the first activity in a joint project of technical assistance by the conveners, aimed at strengthening access to comprehensive post-rape care for survivors of sexual violence. It aimed to sensitize Member States to their obligations under the Maputo Protocol to expand women’s access to emergency contraception (EC) and safe abortion services, and to inspire them to do so by providing information, research evidence, and a platform for discussion.

The meeting deliberations fostered a better understanding of opportunities to broaden access to EC and safe abortion for survivors in the region. Discussions on EC in this regard centered on strengthening EC delivery in the clinical context, decentralizing EC services, increasing community awareness, and overcoming policy barriers. Safe abortion discussions focused primarily on legislation, policy, and integrating these services into existing services for sexual violence survivors. Country-specific action plans were developed to address gaps and weaknesses.

The regional technical meeting concluded with a discussion of practical steps that participants could take to facilitate legal, policy, and program reform with respect to pregnancy prevention and safe abortion in their respective countries. The steps revolved around three mainly areas, namely: establishing an evidence base to inform action; creating forums for discussing the issues; and drafting action points to carry the momentum from the meeting forward. This paper details the proceedings from this regional technical meeting – proceedings that are of interest to the field of sexual and gender-based violence (and reproductive health, more broadly) as challenges faced by countries in implementing the Maputo Protocol are outlined, and evidence-informed and practice-based strategies for addressing these challenges are provided.

## Introduction

The risks of pregnancy and the potential for morbidity, mortality, and grave social consequences are relatively high for female rape survivors [1]. Despite this reality, markedly less attention and investment have been granted to pregnancy prevention and management services – namely, access to pregnancy testing, emergency contraception (EC), and safe abortion – compared to other forms of post-rape care (PRC) [2].

In recognition of this gap, in April 2016, the Population Council, in partnership with the World Health Organization (WHO) and the International Consortium for Emergency Contraception (ICEC), convened a regional meeting in Lusaka, Zambia, geared toward supporting countries in East and Southern Africa in meeting their obligations under the Maputo Protocol (described in further detail below) to expand access to women’s reproductive health services – especially women survivors of sexual violence (SV). Meeting participants included representatives of national Ministries of Health, Ministries of Justice, and/or Non-Governmental Organizations from six countries: Botswana, Ethiopia, Kenya, Malawi, Rwanda, and Zambia. Also in attendance were technical experts from the ICEC, Ipas, Population Council, UN, WHO, and the Zambia Police Service.

These six countries were selected for the following reasons: 1) they were priority countries under the Population Council programs that sponsored the meeting [3], 2) they all had national guidelines on the medical management of sexual violence, 3) the national sexual violence guidelines in these countries all mentioned abortion, and/or the countries had statutes allowing for post-rape abortion, and/or 4) the countries were undergoing legal reforms at the time of the meeting. These first three factors were considered as an indicator of the readiness of the six countries to participate in such a convening, but also of the fact that the countries were far enough along in addressing post-rape care to be able to tackle additional policy or legal issues following the meeting.

### The Maputo protocol

The 2003 Protocol to the African Charter on Human and Peoples’ Rights on the Rights of Women in Africa (the ‘Maputo Protocol’: http://www.achpr.org/instruments/women-protocol/) is the main legal instrument for the protection of the rights of women and girls in Africa [4]. Article 14 of the Maputo Protocol guarantees women’s right to health, including sexual and reproductive health. Women’s rights to sexual and reproductive health include: the right to control their fertility and make decisions regarding when and whether to have children, the right to choose their method of contraception, the right to affordable and accessible reproductive health services, the right to information and education, and the right to be free of violence and coercion. The Maputo Protocol is also the only international instrument that specifically recognizes access to safe, legal abortion as a woman’s human right. Under Article 14 (2) (c), State Parties agree to “protect the reproductive rights of women by authorizing medical abortion in cases of sexual assault, rape, incest, and where the continued pregnancy endangers the mental and physical health of the mother or the life of the mother or the foetus”.

More than ten years since the agreement went into effect, many countries in Africa have still not adopted the necessary laws and policies to effectively domesticate or implement the rights established by the Protocol. A 16-country review of rape survivors’ access to pregnancy prevention and management services in sub-Saharan Africa [1] unveiled a variety of gaps and inconsistencies which contribute to a violation of survivors’ rights and of good medical practice as outlined in international protocols. A major gap highlighted in this study centred on the fact that, with a few exceptions, pregnancy management and safe abortion for survivors do not feature prominently in national sexual violence guidelines in the region. Existing provisions for pregnancy management and abortion also tend to lack detailed, country-specific guidance on laws and procedures that would facilitate access to these services.

Five of the six countries participating in the regional meeting had signed and/or ratified the Maputo Protocol. Nonetheless, in many countries in the region, women and girls still have limited information about, and access to, EC; abortion remains criminalized; and women are effectively prevented from accessing safe abortion services, even in cases where abortion is legally permitted [1]. For survivors of SV, ensuring timely access to emergency pregnancy prevention and safe, legal abortion for unwanted pregnancy remains a critical and urgent challenge [1].

### 2014 Guidance to member states

In May 2014, the African Commission on Human and Peoples’ Rights adopted General Comment No. 2 on Article 14 (1) (a), (b), (c) and (f) and Article 14 (2) (a) and (c) of the Maputo Protocol (http://www.achpr.org/instruments/general-comment-two-rights-women). This General Comment provided interpretive guidance on the specific obligations of States Parties to promote and protect the sexual and reproductive health of women and girls. Adopting a broad, rights-centered interpretation of the Protocol’s requirements, the General Comment made clear that the State obligation to protect and promote women’s reproductive rights extends beyond decriminalizing abortion in a few narrow cases.

The General Comment specifically calls on State Parties to:

■ Domesticate Article 14 (2)(c) to authorize legal abortion in all cases of sexual violence or risk to the life, mental or physical health of the mother or fetus;

■ Eliminate restrictive laws, policies, procedures and practices that impede access to pregnancy prevention services and/or safe, legal abortion, including laws and policies that require women to travel long distances to obtain services, overcome onerous administrative obstacles, present evidence to “prove” rape or incest, or obtain judicial or other third-party approval for the procedure;

■ Implement measures to facilitate access to reproductive health services, and to address social, economic, and institutional obstacles;

■ Allocate sufficient resources to promote and expand reproductive health services; and

■ Create *an enabling legal and political framework* that promotes access to pregnancy prevention and safe abortion for women and girls, including but not limited to, survivors of sexual violence.

### Meeting objectives and structure

The regional meeting was the first activity in a joint project of technical assistance by the Population Council, WHO, and the ICEC, aimed at strengthening access to comprehensive PRC (including EC and safe abortion) for survivors of sexual violence. The principal objectives of the meeting were to:

■ Provide up-to-date information on State obligations under the Maputo Protocol to expand women’s access to EC and safe abortion services;

■ Foster awareness among participants of how pregnancy prevention and management feature in PRC protocols and services in their countries, using WHO guidance as a benchmark for analysis;

■ Support and inspire participating countries to address legal and policy gaps by sharing technical guidance, best practices, and examples of enabling, survivor-centered laws and policies in the region; and

■ Deliver initial technical assistance to participating countries to identify and address key legal and policy issues.

The three-day meeting provided participants with an opportunity to share information and experiences, critically review national laws, policies and guidelines with respect to EC, abortion, and sexual assault management, and develop action plans for addressing specific gaps and weaknesses. Structured around plenary presentations, team exercises, and moderated panel discussions, the meeting drew on the following 4 topics to stimulate discussion and learning:

1. EC and Safe Abortion as Essential Components of Comprehensive SV Care;
2. The current Situation in Participating Countries with Respect to National Guidelines, Laws, Policies and Practice;
3. Opportunities to Expand Access to Emergency Contraception; and
4. Opportunities to Expand Access to Safe Abortion.

These themes are discussed in further detail in the remainder of this paper.

### EC and safe abortion as essential components of comprehensive SV care

A key theme of the meeting was the importance of fully integrating reproductive health into the services routinely provided to SV survivors, and of ensuring that the reproductive rights of survivors are protected in national laws, policies, and clinical guidelines. To this end, a primary emphasis of the expert presentations was on highlighting evidence and/or international guidance (from the Population Council, WHO, and the ICEC) on: the impact of SV and IPV, the importance of comprehensive care for survivors, medical facts on EC, pregnancy management, and access to safe abortion. Subsequent to setting the stage with the evidence and available guidance, the current situation in participating countries (with regard to survivors’ access to EC and safe abortion) became the subject of focus, coupled with discussions around modalities for expanding such access.

#### Global research evidence

##### Impact of SV and IPV on women’s health

The WHO defines intimate partner violence (IPV) as ‘any behavior within an intimate relationship that causes physical, psychological or sexual harm to those in the relationship’ [5]. IPV includes acts of physical aggression, psychological abuse, sexual coercion, and various controlling behaviors.

SV and IPV have significant long-term consequences for women’s physical, mental, and reproductive health [6]. Regional experts presented evidence of the consequences of sexual and intimate partner violence for health outcomes such as unintended pregnancy, infectious diseases, mental disorders, depression, alcoholism, and suicide, as well as complications from unsafe abortion. Unsafe abortion remains one of the most common and avoidable causes of maternal mortality and morbidity in Africa. According to WHO research, 14% of maternal deaths of African women result from unsafe abortion [7]. Meeting participants also noted that women and girls who become pregnant through violence (as well as their children and families) experience long-term social, economic and health consequences when forced to carry an unwanted pregnancy to term. Presenters noted the lack of research on rape- and incest-related pregnancy, and called for more Africa-specific research to provide evidence on both the frequency and impact of such pregnancies on survivors, their children, and families.

With respect to the Maputo Protocol’s grounds for abortion, General Comment 2 states that:

“The Protocol provides for women’s right to terminate pregnancies contracted following sexual assault, rape or incest. Forcing a woman to keep a pregnancy resulting from these cases constitutes additional trauma which affects her physical and mental health...”

In terms of reproductive health consequences, WHO experts noted that women who experience physical, sexual, and/or emotional IPV are at even greater risk of unintended pregnancy (and unsafe abortion) than survivors of non-partner or “one-off” sexual violence. Women experiencing IPV not only are victims of sexual coercion, they also frequently lack control over contraception and family planning (FP) decisions. Nevertheless, most countries that permit abortion in cases of sexual violence do not recognize a need for access to safe abortion for women experiencing IPV, nor is IPV explicitly identified as a basis for legal abortion in the Maputo Protocol (although some cases may be categorized as rape in countries where spousal rape is recognized). According to WHO estimates, IPV survivors are twice as likely to induce abortions (often in settings where there is no access to safe abortion) as other women.

##### Need for comprehensive services

WHO has developed detailed policy and clinical guidance on the care of survivors of sexual assault and intimate partner violence [8]. According to this guidance, female survivors of sexual violence need comprehensive, woman-centered services that meet their physical, psychosocial, and reproductive health needs. Comprehensive PRC includes physical and psychological care, EC, PEP for HIV prevention, treatment for sexually transmitted infections (STIs), collection of forensic evidence, access to safe abortion services, psycho-social counseling, and follow up care.

While post-rape services initially focused on medico-legal aspects of SV, and later, on prevention of HIV, there is a growing consensus on the importance of preventing unwanted pregnancy from sexual assault through provision of EC. This is reflected in national guidelines and PRC protocols: Management of pregnancy from rape, including pregnancy counseling and referral, remains a neglected area of care.

Participants at the regional meeting agreed that prevention and management of pregnancy are essential aspects of comprehensive post-rape care. Care and support to prevent and manage pregnancy should be offered in the context of comprehensive care for the overall management of SV and IPV, rather than in isolation. Effective psychosocial support that includes “first line” response or psychological first aid, is critical [9]. As a WHO expert noted, “Some survivors will need EC, some will need safe abortion, but *all* survivors will need and should be offered ‘first line support’ to address psychological trauma.” Psychological first aid should form part of a complementary, initial mental health and psychosocial response, though it is not the only mental health service that survivors receive as part of comprehensive care.

In addition to offering specialized post-rape care, presenters also identified the health sector as a key entry point for addressing sexual and intimate partner violence more broadly. WHO presenters noted that the majority of women and girls who experience SV/IPV do not report or seek specialized care for such violence. Those that do access health services may enter via delivery points that seem unrelated to SV/IPV care, present with non-violence symptoms, or not reveal that they are experiencing violence. However, most women will receive sexual and reproductive health services at some time in their life. If health workers are sensitized about SV, they can identify women based on the consequent symptoms or conditions, educate women on their rights, provide information to women about their and options, provide referrals, assist in accessing social and legal services, and manage some of the clinical aspects. They can also avoid the harm that results to survivors when health workers do not respond adequately or sensitively to SV. The health sector can also play an essential role in collecting data on the incidence, risk factors and consequences of SV, inform SV policy and program development, and advocate for more integration and prioritization of SV as an issue within the sector.

WHO’s health sector guidelines and tools based on services for women subjected to intimate partner violence or sexual violence, the clinical management of rape survivors, and technical and policy guidelines for safe abortion [10] should be used to strengthen health services and also to advocate for policy change.

##### A technical update on emergency contraception

An ICEC representative delivered a presentation focusing on a technical update on EC. The presentation revealed that EC is one of the safest forms of contraception and works by preventing fertilization, not implantation. Thus, many of the reasons given for not providing EC reflect misinformation and provider bias rather than medical fact. EC is both safe and effective. EC pills are more effective the sooner they are taken after unprotected sex, but can be effective at preventing pregnancy for up to five days. Dedicated EC pills (those packaged and labeled specifically for use as emergency contraception) have no estrogen, have few side effects, and are not associated with infertility, cancer, or stroke. If a pregnant woman takes EC, it will not cause harm to her or to the fetus. It is not a form of abortion. EC pills are safe to be used repeatedly within the same menstrual cycle, and, while not recommended for long-term family planning because other ongoing methods are more effective, there are no restrictions on repeated use.

##### Pregnancy management and access to safe abortion

According to WHO guidelines, survivors who experience rape-related pregnancy should be counseled on their options and referred for abortion where legally available. This guidance is consistent with the Maputo Protocol and statements by the UN Commission on the Status of Women calling for access to EC and safe abortion for sexual violence survivors [11]. Nevertheless, national sexual violence guidelines tend to be silent, or at best, vague on the law with respect to abortion and on counseling survivors who become pregnant regarding their legal options.

Expert presentations also highlighted the health and human right aspects of access to safe, legal abortion, the safety of medical abortion (when performed according to WHO guidelines), and the consequences of unsafe abortion for millions of women globally. According to WHO (in 2008), half of the 44 million abortions obtained annually across the world are unsafe. WHO also emphasized that “highly restrictive abortion laws are not associated with lower rates of abortion – just higher rates of unsafe abortion.” Where states have legalized abortion, abortion-related mortality has declined dramatically (73% in the USA, 91% in South Africa). One WHO presenter reported that, because safe, legal abortion is rarely permitted in Africa, an abortion on the African continent in 2008 was 767 times more likely to end in death than an abortion in the United States [7].

The Maputo Protocol requires that States not only decriminalize abortion in certain circumstances – it also requires that States create an enabling environment that promotes safe abortion care. To achieve this environment, countries must ensure legal access to safe abortion, availability of services, and good quality of care.

Experts also noted that the African Commission has adopted the broader WHO definition of “health” for purposes of the Maputo Protocol:

“When assessing the risks to a pregnant woman’s health, health must be interpreted according to the WHO definition, namely: a ‘state of complete physical, mental and social well-being and not merely the absence of disease or infirmity.”

According to this guidance, any laws, policies or practices that restrict legal abortion to narrowly defined medical grounds do not meet State obligations under the Protocol. The Comment also states that the unavailability of safe abortion is in itself a threat to women’s life and health, due to the high risk of injury and death from abortion by unsafe or illegal procedures, methods and providers [12]. Given the General Comment interpretation, it is clear that safe, legal abortion should be far more available in the region than is currently understood or implemented.

Drawing a parallel between restrictive abortion laws and policies and those relating to HIV, regional experts stressed the need to address unsafe abortion first and foremost as a health and human rights issue. In the case of HIV, punitive laws that criminalized HIV transmission, sex work and drug possession did not reduce HIV, but in fact created barriers to health service access and had a negative impact on utilization overall. To change this, policy makers and health care providers (HCPs) had to “withhold judgment and prioritize access to services.” A similar approach is needed now to reduce unsafe abortion and its negative health consequences.

Participants recognized the difficulty of advocating for EC and abortion with policy makers and in communities, because of the “moral or religious issues” and stigma attached to abortion, rape and incest. WHO presenters encouraged participants to focus on the “health imperative” of preventing unwanted pregnancy and reducing the number of unsafe abortions in Africa, and to frame the debate around reproductive health and SV as a fundamental human rights issue. A Population Council representative also spoke of the human cost of SV and unsafe abortion, calling on participants to be “bold” in the face of resistance, and to champion the “rights of the voiceless.”

### Current situation in participating countries with respect to national guidelines, laws, policies, and practice

A key objective of the meeting was to encourage delegates to look closely and critically at the laws, policies and practices that currently exist in their countries and to compare these with the requirements of the Maputo Protocol and WHO evidence-based guidelines for safe abortion and PRC/IPV. This was achieved through: 1) a Population Council presentation of the key findings from its 2014 study of sixteen African countries, entitled “Access to Emergency Contraception and Safe Abortion Services for Survivors of Rape: A Review of Policies, Programmes and Country Experiences in Sub-Saharan Africa” [1], and 2) a review of WHO guidelines, facilitated team exercises, and sharing of information in plenary.

#### Pregnancy prevention

All of the six countries represented at the meeting have developed national guidelines for the care and management of rape and sexual assault. In each of these guidelines, prevention of pregnancy through EC is included as an essential element of care.

National Post-Rape Care (PRC) guidelines on EC are generally consistent with WHO guidance. However, review of the national guidelines identified some gaps and/or inconsistencies, such as information on determining eligibility for children and adolescents, and the information to be provided to survivors when administering EC. Participants also found that in some cases their guidelines on EC were not fully consistent with the most recent 2013 WHO guidance. Several countries noted the need to update their guidelines to include the following information:

■ Pregnancy testing is not necessary before administering EC. EC should never be delayed or refused if testing is not available;

■ EC should be given to all survivors presenting within 5 days (not 72 h, per prior guidance) who wish to prevent pregnancy;

■ Children and adolescents should be offered EC if they have begun menstruating or have secondary characteristics;

■ EC works by preventing fertilization, not implantation;

■ EC is completely safe and does not interfere with existing pregnancy;

■ EC should be administered as soon as possible within the post-rape care regimen;

■ Dedicated emergency contraceptive pill (ECP) [levonorgestrel (LNG)] may be taken as a Stat dose [13];

■ Children and adolescents should be offered EC if they have begun menstruating or have secondary characteristics;

■ EC should be offered regardless of where the woman is in her menstrual cycle, as it is difficult to predict the timing of ovulation;

■ Prophylactic administration of anti-emetics is not needed when patients are given dedicated ECPs (LNG);

■ Survivors should be offered a longer-term contraception option after being given EC.

WHO and ICEC guidelines recommend the use of dedicated ECP (1.5 mg of LNG) for emergency contraception. This is not always possible, however, as many countries in Africa do not stock dedicated ECP in public health facilities, or they experience frequent stock-outs. Participants therefore noted the need to include more information for health providers in national and clinical guidelines on how to use regular contraceptive pills (progestogen-only contraceptive pills or combined oral contraceptive pills) for EC. “Customizing” guidelines to reflect the specific brands and regimens available can also be helpful for providers, but may require frequent updating. WHO representatives at the meeting recommended that these details be provided in training or clinical instructions rather than the national guidelines.

Discussions revealed that the majority of countries represented at the meeting do not stock dedicated ECP (levonorgestrel) in their public health facilities or pharmacies in practice. Kenya is an exception in this regard, as all public facilities are provided dedicated ECP, in part by donor organizations. Dedicated EC is also readily available from the private sector. Other countries, such as Rwanda, provide dedicated EC in specialized PRC facilities such as “one-stop centers,” however most public clinics only have regular contraceptive pills to use for EC. In some countries, participants stated that dedicated EC is available in public sector pharmacies and family planning clinics but may not be stocked in emergency rooms or other places where PRC services are provided. In Botswana, dedicated ECP is only available from private pharmacies - public facilities, including those designated for PRC- are currently limited to offering combined oral contraceptive pills. In Ethiopia, EC is available in public facilities - according to one participant, no prescription is required, there is no age restriction for access and even men can come in and ask for it for their wives and girlfriends.

According to participants, EC is available over the counter in pharmacies without a doctor prescription in three of the six countries at the meeting - Ethiopia, Kenya, and Malawi [14]. In the other three, it may be obtained at private pharmacies, but a prescription is required [15]. According to ICEC, there is no medical reason why EC should not be available over-the-counter. Requiring a prescription reduces access because many women, especially young, poor, and rural women, do not have the time, opportunity or resources to see a doctor or to access EC from a clinic during regular hours. For women in urban areas, over the counter EC substantially increases opportunities for access, without having to see a doctor or report to a public facility offering post-rape care. Access is even more challenging for women and adolescents in rural areas, where private-sector EC is not always an option: there are fewer pharmacies, pharmacies may not carry it, it is too expensive, or there are no doctors at the clinics to prescribe it.

The findings from the Population Council study regarding the policy framework for EC were generally consistent with the presentations by country teams, particularly in terms of coverage of EC in national PRC guidelines. The study found that national guidelines consistently identify pregnancy prevention as an essential element of sexual violence care, and all countries with national guidelines included provisions on EC. Population Council also found, however, gaps and inconsistencies in the EC provisions, as well as a disconnect between PRC guidelines and national guidelines for reproductive health and family planning. The study found, for example, that although many RH policies contained general provisions on EC, EC was not given much attention compared to other FP methods. Moreover, most national RH/FP guidelines tended to be vague or silent on the specific reproductive health needs of rape and incest survivors and the need to integrate EC into both PRC and regular RH/FP services in order to ensure that all eligible survivors have access to EC. During the regional meeting, several countries identified the need to align the language in their RH/FP guidelines with the language in the PRC guidelines, particularly in terms of offering EC to all SV survivors and the information to give patients prior to administering EC.

#### Key challenges to EC access

##### Participants’ perspectives on barriers to EC access

During the meeting, participants were asked to identify what they felt were the major challenges to EC access. The results of this group exercise were then analyzed and summarized by an ICEC expert as follows:

■ At the Personal Level, low awareness of EC among survivors and the public generally was the most frequently identified obstacle, followed by stigma associated with rape or pre-marital sex;

■ At the Provider Level, lack of knowledge regarding how EC works and how to administer it correctly, was the primary barrier identified by participants, followed by provider biases, misconceptions and attitudes toward EC and survivors;

■ At the Policy Level, participants identified supply chain management and logistics as the biggest challenge, resulting in frequent “stock-outs” of EC products in public facilities.

The findings closely mirror results from the Population Council study on access to EC for survivors in the region [1]. This study highlighted low awareness of EC among survivors and other community members; failure to report or seek health services following rape; ‘late reporting’ (reporting past the timeframe for EC); providers’ unfamiliarity with their national protocols or the details of how to administer EC, including to rape victims.

#### Pregnancy counseling, management, and safe abortion care

WHO’s most recent guidance on *Responding to Intimate Partner Violence and Sexual Violence Against Women* (2013) states that if a survivor presents after the time required for EC, if EC fails, or if she presents with a pregnancy as a result of rape or incest, she should be offered safe abortion in accordance with national law. Survivors who decide to maintain the pregnancy should be advised of all available options (including adoption and foster care), and referred to pre-natal care.

WHO’s guidance on Safe Abortion [16] further states that women who are pregnant as a result of rape:

“have a special need for sensitive treatment, and all levels of the health system should be able to offer appropriate care and support. Standards and guidelines for provision of abortion in such cases...should not impose unnecessary administrative or judicial procedures such as requiring women to press charges or identify the rapist ... and should ideally be part of comprehensive standards and guidelines for the overall management of survivors of rape.”

Further:

“[p]rompt, safe abortion services should be provided on the basis of the woman’s complaint rather than requiring forensic evidence or police examination. Administrative requirements should be minimized and clear protocols established for both police and health-care providers as this will facilitate referral and access to care” [16].

Similar standards for safe abortion for survivors are required under the Maputo Protocol, as articulated in the African Commission’s General Comment 2.

Consistent with the findings of the Population Council’s 2014 study [1], the meeting confirmed that few countries in the region provide safe abortion for sexual violence survivors as required by the Maputo Protocol. With the exception of Ethiopia, none of the six countries represented at the meeting had clear guidelines on abortion for sexual violence survivors. Ethiopia was also the only country of the six participating in the meeting that features access to safe abortion in its national protocol as an essential element of post-rape care.

Like Ethiopia, Botswana and Rwanda have national laws permitting legal abortion in the case of rape and incest, as well as in cases where the pregnancy presents a risk to the life or health of the mother, or in cases of severe fetal abnormality. However, of these three countries, only Ethiopia was found to have an “enabling” legal and policy framework in which survivors of sexual violence are referred for abortion if requested, and can actually access safe abortion in practice. Due to judicial and/or administrative requirements contained in the law or implemented in practice, safe abortion is almost as inaccessible to sexual violence survivors in Botswana and Rwanda as in countries where abortion is illegal.

In two of the participating countries - Kenya and Zambia - the legality of abortion for sexual violence survivors is unclear, and therefore tends to be interpreted narrowly. Neither country explicitly allows legal abortion in case of rape or incest. However, both countries have adopted provisions (in the constitution and by statute, respectively) that allow for legal abortion to protect the life and health of the mother. If interpreted expansively, according to General Comment 2, these current exceptions are sufficient to allow legal abortion where pregnancy is the result of sexual violence. In practice, however, abortion is not generally offered to SV survivors and is considered by most to be a criminal offense. Zambia has reportedly initiated a process to update its 1972 termination of pregnancy law to meet the requirements of the Maputo Protocol. At present, however, only child survivors of sexual violence are expressly permitted to obtain abortion (per an amendment to the penal code). The procedure for accessing abortion in these cases remains unclear.

In Malawi, participants explained that abortion remains illegal except to save the life of the mother, and that in practice, this provision is interpreted narrowly. Pregnancy is not addressed in the national PRC guidelines, and survivors of sexual violence who become pregnant are not offered abortion as part of post-rape care.

Participants from Malawi reported that the Malawi Law Commission has proposed amended legislation that would broaden the law to include all the exceptions required by the Maputo Protocol, including a specific rape/incest exception. However, little progress has been made in enacting the law since it was proposed in 2009.

In contrast, participants from Ethiopia explained that according to their law, safe abortion is available in all cases required by the Maputo Protocol, as well as to any young woman under the age of 18 by virtue of her minority status. Ethiopia is also one of the few countries in the region that has developed national clinical and policy guidelines for safe abortion care, establishing a clear policy framework for safe abortion in addition to clinical protocols for practitioners [17]. Of note was the fact that Ethiopia’s law, consistent with WHO guidelines and General Comment 2, allows abortion services to be provided at the woman’s request, if her reason is one of the reasons allowed by the statute. She is not required to provide “proof” of eligibility or obtain a doctor or judge’s permission in order to access the service. As one presenter explained, “If the woman says she was raped, that is enough ... Nothing further is required.”

#### Treatment of pregnancy and safe abortion in national guidelines

As with EC, participants at the meeting conducted an in-depth review of provisions in their national PRC guidelines regarding pregnancy testing, counseling and management. According to WHO guidelines, pregnancy testing is not required prior to giving EC, as it will not harm an existing pregnancy and any pregnancy from rape could not be detected at that stage. Rather, pregnancy testing should be conducted at the recommended two-week follow up visit if the survivor is at risk for pregnancy, or at the first visit if a survivor presents too late for EC.

The participant review confirmed that pregnancy testing features in all of the national PRC guidelines. All six recommend a baseline pregnancy assessment [18] (although not required by WHO guidelines) and re-testing for pregnancy at some stage after the survivor’s initial visit (ranging from two weeks to six weeks). However, participants generally agreed that their guidelines could be strengthened by specifically addressing the needs of “late reporters” and by providing clear guidance and an adapted flow-chart for these patients. For example, participants noted that late reporting survivors should be tested for pregnancy at their initial visit and managed accordingly, regardless of when they report for care. Participants also pointed out the need to ensure that survivors who receive EC are routinely tested for pregnancy after two weeks - per WHO guidelines - rather than waiting for a 4 to 6 week follow-up. At minimum, they noted that survivors should be informed to return immediately for testing in the event of a missed period.

With few exceptions, the review showed also that pregnancy counseling and management does not feature prominently in national PRC guidelines. Where discussed at all, national provisions tend to be vague or stated in general, boilerplate terms, with little in the way of detailed guidance or country-specific information. This is particularly true with respect to access to abortion. In countries where abortion after rape is not specifically allowed, the protocols are silent. In others, the provisions are unclear and provide little guidance to health care providers or patients in terms of their rights or options. Ethiopia’s national PRC guidelines [19] provide the clearest guidance on pregnancy, focusing on the following needs of survivors of rape: emotional support to foster understanding of their options if pregnant, antenatal care for those that opt to keep the pregnancy, adoption and foster care as options, and abortion services.

Most participants agreed that their guidelines could be strengthened with respect to pregnancy counseling and management, although some noted the difficulty of doing so before the laws and policies on abortion in their countries are changed or clarified. Others noted, however, that there was room for clearer and more favorable interpretations of existing laws to be incorporated into PRC guidelines. Such a process would be helpful in expanding survivors’ access to safe abortion within the framework permitted by law. According to one expert, “If the law is vague, then we need to play a role in interpreting them. If interpretation is at the discretion of health care providers, then we need to empower them to interpret the law more expansively.”

With respect to counseling, WHO experts noted the importance of providing accurate and evidence-based information to women to allow them to make their own decision regarding their pregnancy. Pregnancy counseling should be client-centered and non-directive, focused on the woman and her needs rather than the counselor’s personal views and opinions.

Participants and experts also noted the importance of identifying *specific* referral services for survivors who become pregnant, particularly at facility level, and ensuring that survivors are able to access them [20]. Whether for abortion, adoption/foster care, further counseling and support, or PMTCT/pre-natal care, identifying available services and putting formal referral arrangements in place, can help ensure that survivors receive the care they need.

#### Key challenges to safe abortion access

##### Legal provisions

The most significant challenge to safe abortion is the legal and policy framework in the majority of countries represented at the meeting. As mentioned previously, with the exception of Ethiopia, none of the participating countries currently have a clear and enabling policy allowing safe abortion for survivors of sexual violence – not even in countries such as Botswana and Rwanda where abortion after rape or incest is legally permitted. In some countries, the challenge lies in vague and/or conflicting laws or the narrow interpretation of these laws. In others, the law requires onerous third-party approval processes to obtain an abortion, which significantly impede timely access to the procedure. In Zambia, for example, the law requires three doctors, including one specialist, to give their approval before an abortion may be provided. In a country with very few doctors, this provision is prohibitive, particularly in rural areas. Experts also noted with concern how few countries in Africa allow abortion at the request of the survivor, and instead place the decision-making in the hands of third parties.

##### Lack of policy guidance

Few countries in the region have developed clear policy guidance interpreting existing law or setting out the procedures for accessing safe, legal abortion. The result is that few health care providers, police, or members of the public understand what is and is not legally permitted, or what survivors must do to obtain legal abortion services. Concerns about criminal penalties cause practitioners to err on the side of caution, refusing abortion in situations arguably permitted under the law. Moreover, in the absence of clear policy, too much discretion rests with the individual health care providers or facilities, and the “policy” becomes what is done in practice, rather than the other way around. During the meeting, it became evident that some countries are interpreting the law more strictly than necessary- in practice, imposing evidentiary burdens and approval processes above and beyond the requirements of the law in the absence of a clear policy framework. An example is Botswana where many hospitals require a panel of doctors to approve abortion for SV survivors even though not required by law. Participants also stated that, in practice, pregnant survivors seeking abortion must prove not only that they were raped (i.e., by providing evidence of a police report or medical exam), but that they became pregnant because of rape (i.e., they were not sexually active before or after the sexual assault) before they can be referred for abortion. Even children, who are by definition victims of defilement/statutory rape, must provide evidence and go through these approval procedures. Participants noted that while all of the participating countries have protocols on post-abortion care (PAC), only Ethiopia has a national health policy on safe abortion. Rwanda reported that it is in the process of developing guidelines in connection with its new abortion law.

##### Low awareness

Limited awareness of abortion laws in the country or confusion regarding their interpretation can result in survivors not accessing abortion even where it is legal under the law. As the primary “gate-keepers,” health care providers play a critical role in informing women of their rights and the options available to them. It is important, therefore, that HCPs are well-trained to provide accurate, un-biased information on abortion and abortion procedures, and to understand the law and Ministry of Health policy regarding the right to access. As one participant explained, “Moving forward, there is a critical need to interpret the law and educate the public so that people understand what the law says. At the moment, lawmakers don’t know. Service providers don’t know. And the people who need these services don’t know. Most assume [abortion] is illegal even when it’s not.”

##### Capacity and training

Capacity to provide accessible, high quality abortion services is currently limited, even in countries where abortion is legally available. In order to expand access to abortion, participants noted the need to increase the number of trained providers and facilities offering the procedure, and to more fully integrate safe abortion into post-rape care. Training also needs to address moral objections to abortion.

Of additional concern are laws which only allow doctors to perform abortions, or require abortions to be performed only in hospitals. These create unnecessary barriers to access in areas where doctors are rare and hospitals are far away. WHO guidance is clear that abortion should be accessible at all levels of the health care system and that lower level HCPs can be trained to safely provide medical and non-surgical abortions.

##### Age of consent

A few participants expressed concern that minors are not able to access abortion without parental consent, even where they are above the age of consent for sex. This creates a barrier, because many do not want to reveal sexual violence or sexual activity to their parents and wait until it is too late to obtain abortion safely. Ethiopia was the exception among participating countries, as minors are allowed to access abortion without consent.

##### Legal challenges by conservative/religious groups

In several countries, “pro-life” groups have vigorously opposed expansion of abortion rights. In Kenya, there is currently a case in the Supreme Court challenging the dissemination and implementation of health ministry clinical protocols on safe abortion. Participants from Zambia also reported that a proposed constitutional provision stating that life begins at conception could effectively eliminate legal abortion in the country, at the same time that key stakeholders in Zambia are working to expand access to safe abortion.

### Opportunities to expand access to emergency contraception

To date, efforts to strengthen access to EC for survivors of sexual violence have focused primarily on including EC in the package of PRC services offered in health facilities and one-stop centers. As reflected in the national protocols, EC is increasingly recognized as an essential component of comprehensive post-rape care. The challenge remains, however, that in many settings a majority of sexual assault survivors do not report to law enforcement or seek facility-based treatment. For these women and girls, knowing that EC is an option to prevent pregnancy and being able to obtain it through other avenues is critical.

During the meeting, participants explored various ways to improve access to EC both within and outside the clinical context. Discussion focused on four main areas: strengthening delivery of EC in the clinical context, decentralization of EC services, increasing awareness in the community, and overcoming policy barriers.

#### Enhancing clinical contexts

##### Training

Participating countries need to ensure that EC is integrated into HCP training on family planning (FP) and SV. It should be part of the regular curriculum for pre-service as well as in-service training. The training should focus on the safety, side effects, and mechanism of action of EC, along with how to provide EC, including how to use regular contraception pills for EC. This is a critical component since many facilities do not yet supply or have a regular supply of dedicated EC pills. As one participant explained, “Women should not be turned away because dedicated EC products are not available.”

##### Access to products

Countries need to strengthen access to supplies and management of the supply chain. Some countries procure dedicated ECPs while others do not. Even in countries where the government procures it, there may be other barriers to ensuring its availability. In Zambia, for example, EC is not included on the Essential Medicines List. As a result, EC is not automatically sent to lower level facilities such as clinics, and even dedicated SV facilities experience stock-outs. Countries need to procure EC and then ensure that it is distributed regularly to every health care facility without the need to special order. Countries should also be sure to include EC on facility-level order forms and reporting forms to request EC and keep track of EC usage. According to ICEC, including EC on such forms can be very helpful in improving country-level procurement and distribution.

##### Availability 24/7

Saying EC is available in the “public sector” is not sufficient, as that often only means that EC can be obtained from a FP clinic or pharmacy during regular business hours. Given the narrow timeframe for EC and the impact of timing on efficacy, women, and especially SV survivors, need to be able to access EC at any health facility - from local clinic to emergency room, on weekends and after hours, and without having to travel long distances to regional hospitals or specialty centers. Facilities also need to ensure that the staff on duty can provide EC (and have access to ECP) even if a doctor is not available.

##### Facility-level protocols

To improve access at facility-level, facilities offering care to sexual violence survivors should incorporate the following into their service protocols:

■ EC should be provided as early as possible in the treatment flow.

■ EC must be available and accessible to HCPs 24/7.

■ EC must be physically available *in the place where SV services are provided*, including during off hours, so that patients do not have to wait at the pharmacy, travel to other wards or off-site facilities, or wait for services to open for business in order to obtain EC.

■ EC should be given to patients free of charge.

■ Survivors should not be required to report to police in order to receive EC.

■ Provision of EC should be included in staff monitoring/evaluation/supervision and oversight.

##### Youth-friendly services

Countries need to make EC more accessible to young people, including through youth-friendly services at local clinics. In Botswana, for example, some public clinics have created a “youth corner” staffed with younger nurses (male and female), which has improved access to EC in this context. Young people also need to have access to more complete and accurate information about sexuality, contraception and SV to counteract the stigma and “culture of silence” around EC and SV. HCPs can play a role in promoting more open discussion around these issues, as can creative use of social media.

##### Confidentiality

FP protocols for EC distribution must be sensitive to women’s privacy concerns and protect their confidentiality. In Kenya and Botswana, for example, women requesting EC from public sector clinics have to record their names in a register, as with other forms of FP. Given the circumstances under which women seek EC, countries should amend these requirements to permit recording of EC distribution without identifying information so as not to inhibit women and girls from seeking EC from the public sector, especially after rape.

##### Sensitization

The health profession in general needs to be more open and sensitized regarding SV, sexuality, reproductive health, and rights, “so that people - and especially young people - can go and get help, feel free to talk, and ask questions without judgment,” including, but not limited to, questions about EC.

##### Decentralization of services

A second way to improve access is to make EC available in a broader range of public and private settings, and to assure access, regardless of whether sexual violence is reported. Making EC widely known and available can help survivors access EC – especially those who choose not to report or seek services at a health facility.

Recommendations from the meeting include:

##### Over-the counter access/private pharmacies

Countries could enter into public/private partnerships with pharmacies to improve information on the safety of EC and the appropriateness of OTC purchase of EC at pharmacies and drug stores. Such actions would help enhance access to services. In Vietnam, for example, certain private pharmacies have been selected and trained as designated “Youth-Friendly Pharmacies” [21] to improve access to young people.

##### Make EC available to SV survivors at the first point of contact

Participants from Zambia indicated that Zambia-based research showed that 90% of rape survivors who reported their rape incident, went first to the police, not to health care facilities. In response, Zambia implemented a pilot project to provide EC at police stations through the Victim Support Unit (VSU) [22]. These officers were trained to provide EC, provide information about PRC, and refer survivors for further services. The project also included a community outreach component in which officers went out to communities and told people about the services available. As a result of the project, the number of women reporting to police, obtaining EC, and being referred to hospitals for comprehensive PRC all increased. It also helped improve the public’s perception and attitudes toward police. This role of EC provision to survivors by VSU police officers is now also stipulated in Zambia’s national guidelines for the management of survivors.

##### Make EC available in schools and workplaces

In Botswana, for example, EC is available in clinics based at tertiary educational institutions, as well as some public sector workplaces such as the Botswana Defense Force.

*Use Community Health Workers/Volunteers to expand access to EC, especially in rural communities.*

In Zambia, the ministry is currently using community health workers (CHWs – also referred to as ‘community health volunteers in some countries) to expand access to health services, especially in rural areas where there are few clinics, few professional nurses, and no doctors. If properly trained, CHWs could also be used to provide family planning services and EC. In Kenya, community health volunteers are already allowed to distribute regular contraceptive pills. At minimum, these same volunteers should be trained on how to use regular contraception for EC. With additional training, community health volunteers could also distribute dedicated ECP and refer women in their communities for longer-term family planning services and/or comprehensive IPV or SV care [23].

Some participants noted a potential downside of decentralizing services and increasing access to EC outside of health facilities or specialized PRC centers, namely that SV survivors may choose to take EC without obtaining other essential services, such as psycho-social counseling and PEP to prevent HIV infection. As a result, some participants argued that efforts should focus on strengthening access to comprehensive PRC, and identifying and addressing the barriers that prevent survivors from seeking health services. Some also expressed concern about community workers providing services to survivors of violence, noting sensitivity, ethical and privacy concerns. According to one expert, community health workers can be used to raise awareness, but are not qualified to provide counseling and other services to survivors, and risk causing further harm. There was consensus among participants on this point, however- if community workers are allowed to provide EC, it is essential that they be trained to provide correct information, protect survivor privacy and confidentiality, and refer survivors to health care facilities for comprehensive services. As one participant stated, “EC should not be stand-alone response.”

#### Increasing awareness

Participants in the meeting agreed that low awareness of EC is a major challenge to increased access. According to DHS data, less than 40% of women have ever heard of EC in five of the six countries that participated in the meeting (Kenya is the exception). Those that have heard of EC may not know how it works, the timeframe for using EC or how and where to access it. Increasing public awareness of EC, and SV services generally, is thus an urgent need. SV services should include a community awareness and outreach component so that survivors will be informed of the services available to them and encouraged to access those services in a timely fashion. Some specific recommendations (made based on existing approaches) and suggestions were outlined to help promote EC awareness:

##### School-based education and outreach

Countries need to integrate information about EC and SV into the regular health education curricula. Various role players can also conduct outreach in schools. In Zambia, for example, the police have a “school zone” unit, which goes out to schools and raises awareness about SV and services available to SV survivors, including EC.

##### Integrate EC into FP visits

Raising awareness about EC should be part of regular family planning and counseling. If EC is explained during routine appointments, women will be more aware of their options if and when they or someone they know needs EC. Integrating EC into FP counseling and information materials is also important for other reasons. As explained by ICEC, there is an urgent need to “set the facts straight” about EC, as many people have been given inaccurate information or have misconceptions about EC. Women also need to understand that EC is not the most effective method of birth control. They need to be aware of its limitations and the importance of regular contraception/FP strategies for longer-term protection against pregnancy and HIV. Government from across sectors, including but not limited to health, should team with civil society to raise awareness of EC, available SV services, and the need for a multi-sectoral response to address sexual violence and IPV.

##### Advocacy and information campaigns using multiple approaches

In the DRC ICEC is working on a project with Population Media Center. PMC produces soap operas and radio shows that are very popular with women. ICEC is working with PMC to incorporate EC-related messages into the program story lines to raise awareness about EC. This is an example of how large audiences might be reached by leveraging existing media platforms.

In Kenya, LVCT Health (a non-governmental organization) has partnered with Safaricom (a leading communications company) to provide a caller information line focused on reproductive health issues. According to members of the Kenya delegation, this strategy has been very effective and benefited thousands.

Community health workers can also be used to disseminate information about EC and SV services, particularly in rural areas. Traditional leaders are also important in rural culture and are very influential in their communities. As one panelist explained, “If we can train the leaders and get them on board, the people will get the information.”

##### Social marketing

Countries need different approaches for rural and urban populations and various age groups. Strategies must be appropriate and effective for the target audience. As one panelist explained, “Working with chiefs is a good strategy in rural areas but a different approach is needed to reach young people in the cities.” Marie Stopes, for example, recommends creating youth-friendly spaces outside the clinical setting, where young people can obtain information and speak openly about sexuality and reproductive health. Social media and mHealth campaigns are also effective ways to reach young people, provide information and improve uptake of services.

The social marketing sector is a natural fit for EC: Research shows that women like to buy EC from pharmacies and often are willing to pay for it. Social marketing programs have effectively distributed EC in a number of countries, including a large program in Ethiopia; yet as of 2012, only a third of social marketing family planning programs in Africa included EC.

#### Addressing policy barriers

##### Improve supply and distribution of dedicated ECP

As discussed above, there is a need to improve supply-chain logistics and management so that health care facilities can properly estimate their needs and ensure uninterrupted supply. Including dedicated EC on the Essential Medicines List is an important step, and ensures that all health facilities, including local clinics, have a supply of ECP. To the extent possible, governments should procure and distribute dedicated EC products; however, where dedicated ECPs are not available, protocols should be in place for supplies of regular oral contraception to be used as EC.

##### Over-the counter access

EC should be available over-the-counter without a prescription and without restrictions as to age.

##### Eliminate reporting requirements

In policy and/or in practice, many countries require survivors to report to police and/or provide a police report before they can access PRC services – including free EC – at public health facilities. These policies should be reviewed and amended, and staff at facilities trained, to ensure that survivors of sexual violence and IPV can obtain essential health services without reporting to law enforcement.

### Opportunities to expand access to safe abortion

Expanding access to safe abortion is a multi-step process which requires an enabling legal and policy framework, awareness of rights, availability of services and trained service providers, and quality of care. Given the limitations of the current legal framework in most countries, discussions around access focused primarily on legislation and policy, as well as on integrating safe abortion more specifically into services for SV survivors.

#### Legislation

In terms of legislation, several participating countries that have not updated their abortion laws post-Maputo identified a need to enact legislation or amend existing statutes to bring them into compliance with Article14(2)(c). At a minimum, the Maputo Protocol requires that national laws authorize legal abortion in all cases of rape or incest, risk to the life, mental or physical health of the mother, and serious fetal abnormality. Some countries in the region also allow abortion on other grounds, such as socio-economic circumstances (Zambia) or the age of the pregnant woman (Ethiopia).

Countries in the region that already permit abortion in certain circumstances need to revisit existing or proposed legislation in light of the new interpretive guidance provided by the African Commission General Comment 2. In particular, the General Comment states that countries should eliminate or revise any provisions in the law that may impede access to safe, legal abortion where authorized, or that run contrary to the idea of an “enabling environment.” These include, for example: evidentiary requirements; third-party authorization requirements and procedures; and overly restrictive requirements regarding how and where abortions may be performed, and by what level of HCP.

General Comment 2 also adopts a broad definition of the terms “life” and/or “health” of the pregnant woman, per the WHO definition of “health,” which arguably encompasses a much wider range of circumstances for legal abortion beyond those currently permitted by law or in practice.

#### Interpretation/implementation of existing law

Beyond enacting or amending legislation, opportunities also exist for line ministries to interpret and implement existing law in a way that is more expansive and favorable to SV survivors. For example, countries can make clear through policy and/or policy directives that any pregnancy of an underage girl is by definition statutory rape, and therefore automatically subject to the provision allowing abortion in the case of rape or incest without the need for any further evidence or approval process. Countries can also allow SV and IPV survivors to access legal abortion on the grounds that it is necessary to protect their mental and physical health. Such a provision is consistent with WHO guidance and General Comment 2, as well as with the British case Rex v. Bourne. Countries can also reduce administrative and evidentiary burdens by allowing survivors to obtain a safe abortion based on their statement alone, or in conjunction with a statement by a SV service provider or survivor advocate, without having to provide a police report or medical evidence to prove sexual violence or pregnancy from sexual violence. Similar opportunities for interpretation through policy and program measures exist in terms of other allowable grounds for abortion, including reasons of maternal or fetal health. As a corollary to all of this, working to address underlying social norms that work against prohibit women’s access to abortion services would be critical.

Where the statute contains specific requirements, stakeholders can work together to design and implement more survivor-friendly procedures while still complying with the law. In Rwanda, for example, the law requires a “court order” for abortion in the case of rape and incest, but does not dictate any particular process for obtaining one. Up until now, survivors have had to navigate the court process alone and very few have succeeded in obtaining court approval. In response, members of a sexual and gender-based violence working group in the country are looking at possible ways to facilitate the process for survivors, i.e. by allowing the HCP or police officer at one-stop facilities to submit a request to the court on behalf of the survivor and obtain court approval without the survivor having to appear or present evidence in court. They are also looking at ways to streamline the approval process required for abortion based on a health condition.

Participants also discussed the possibility of bringing a test case to challenge the narrow interpretation of existing law. In Malawi, for example, attorneys are considering bringing a test case based on Rex v Bourne – a British case that broadly interpreted the same penal code provision on abortion that still exists in many of the commonwealth countries. In this case, the court found that protecting the “life of the mother” included protecting physical and mental health, and that terminating an unwanted pregnancy from rape was justified to protect the mother’s health. The court thus held that a girl who had become pregnant from sexual assault was entitled to legal abortion, even where the law allowed abortion only to save the life of the mother. Several legal experts, including those from the Center for Reproductive Rights, have argued that in the absence of conflicting law, Rex v Bourne provides a legally valid interpretation of the abortion law in countries such as Malawi, Tanzania and Uganda.

#### Awareness of and access to quality services

Experts also pointed to State obligations in the Maputo Protocol and in General Comment 2 to ensure that rights protected by law are promoted and fulfilled in practice. This requires not only that abortion is authorized in the circumstances allowed by law, but that people are informed of their rights and have access to quality services in the communities where they live.

#### Integration of safe abortion into national guidelines

In terms of national guidelines, participants agreed that PRC guidelines need to incorporate clear and accurate information on the law and women’s right to access abortion in the context of rape and incest, including statutory and marital rape. The information should provide clear guidance for survivors and for HCPs on what is and what is not legally possible and how survivors can access available services. The guidelines should also integrate clear guidelines for non-directive pregnancy counseling, including available options and referral mechanisms to facilitate access to available services.

### Moving forward

The regional technical meeting concluded with a discussion of practical steps that participants could take to facilitate legal, policy, and program reform with respect to pregnancy prevention and safe abortion in their respective countries. The steps revolved around three areas:

- establishing an evidence base to inform actions within countries;
- creating forums for discussing the issues; and
- drafting action points to carry the momentum from the meeting forward in individual countries.

#### Establish an evidence base

Panelists agreed on the need to conduct research and establish an evidence base to inform policy decisions, improve programming, and support advocacy efforts. As one panelist stated, presenting evidence from research helps to frame the issue as a medical one. It can also be used to “start the conversation” with others. Reliable health data - including on maternal mortality and other consequences of unsafe abortion – is needed to drive the discussion around abortion reform and counter “emotive arguments” made by pro-life advocacy groups.

With respect to abortion, panelists recommended conducting an impact assessment of current laws and policies, as was undertaken in Rwanda. In Rwanda, facilities began collecting data on the number of survivors becoming pregnant from rape and the number of rape survivors who were able to access legal abortion. From this study, Rwanda learned that since the new law came into effect allowing abortion in cases of sexual violence (including statutory rape), only one survivor was able to obtain an abortion. This evidence is now being used to advocate for a change in the way the law is interpreted and implemented.

Evidence can also be used to make changes on the ground, even where change is slow at the legislative and policy level. In Zambia, for example, Population Council is collecting evidence on such issues as quality of abortion care and the availability of medical abortion. Population Council also called for more research on the impact on women of unwanted, rape-related pregnancy to inform advocacy in this area.

#### Create forums for discussing issues

In Kenya, Zambia, and Malawi, interested stakeholders have formed working groups specifically focused on expanding access to safe abortion, the problem of unsafe abortion and the lack of quality abortion services. In Kenya, members of the Safe Abortion Working Group are primarily from the private sector, but the group is led by the maternal health division of the Ministry of Health. In Zambia, the Safe Abortion Action Group is a partnership between non-governmental organizations and the Ministry of Health. Malawi also has established a Coalition for Prevention of Unsafe Abortion (COPUA). In contrast to other technical working groups dealing with family planning and reproductive health more generally, having a forum specifically for abortion has facilitated frank discussion and helped to focus the group’s efforts. It has also helped build partnerships among stakeholders and between the private and public sector.

#### Draft an action strategy

Final discussions from the meeting focused on developing country-specific action points to improve access to EC and safe abortion services, particularly for survivors of sexual violence. Key action points from participants included:

■ Increasing public education around pregnancy prevention, EC, safe abortion, and accessing available SV services (while framing these as public health issues)

■ Updating SV guidelines and training to reflect most recent WHO guidance

■ Integrating EC and safe abortion issues into existing SV and FP training, protocols, and documents

■ Decentralizing EC services to expand access to young people and rural communities

■ Expanding public sector procurement and distribution of dedicated ECP in health facilities

■ Improving forecasting and management of EC supplies

■ Educating line ministries and policy makers regarding their obligations under Maputo, as interpreted in the African Commission’s comments

■ Advocating for favorable interpretation of existing abortion law, or using Ministry of Health policy to interpret and clarify the law relating to safe abortion rights and procedures

■ Organizing partners and like-minded individuals to advocate for reform and address gaps in services

As a follow-up to the meeting, the Population Council, WHO, and the ICEC committed to providing technical support to selected participating countries to facilitate distinct action points as outlined in the draft action plans to help bring countries’ national laws, policies, and/or programming into compliance with the Maputo Protocol and with WHO technical guidelines for safe abortion and comprehensive post-rape care.
