# Correction to: Harmonizing national abortion and pregnancy prevention laws and policies for sexual violence survivors with the Maputo Protocol: proceedings of a 2016 regional technical meeting in sub-Saharan Africa

**DOI:** 10.1186/s12919-018-0103-3

**Published:** 2018-06-05

**Authors:** Jill Thompson, Chi-Chi Undie, Avni Amin, Brooke Ronald Johnson, Rajat Khosla, Leopold Ouedraogo, Triphonie Nkurunziza, Sarah Rich, Elizabeth Westley, Melissa Garcia, Harriet Birungi, Ian Askew

**Affiliations:** 1 Public Counsel, 610 South Ardmore Avenue, Los Angeles, CA 90005 USA; 2 Population Council, P.O. Box 17643-00500, Nairobi, Kenya; 3 Department of Reproductive Health and Research, World Health Organization, Avenue Appia 20, 1211 27 Geneva, Switzerland; 4 Regional Office for Africa, World Health Organization, BP 06 Brazzaville, Republic of Congo; 5 Women’s Refugee Commission, 15 West 37th Street, 9th Floor, New York, NY 10018 USA; 6 International Consortium for Emergency Contraception (hosted by Management Sciences for Health), 45 Broadway, Suite 320, New York, NY 10006 USA

## Correction

After publication of the original article [1], it was noticed one of the author’s Given Names was misspelled. Sarah Rich’s name was misspelled as Sara Rich. The author’s name is correctly presented in the author list of this correction.
